# Molecular alterations and PD-L1 expression in non-ampullary duodenal adenocarcinoma: Associations among clinicopathological, immunophenotypic and molecular features

**DOI:** 10.1038/s41598-019-46167-y

**Published:** 2019-07-19

**Authors:** Jiro Watari, Seiichiro Mitani, Chiyomi Ito, Katsuyuki Tozawa, Toshihiko Tomita, Tadayuki Oshima, Hirokazu Fukui, Shigenori Kadowaki, Seiji Natsume, Yoshiki Senda, Masahiro Tajika, Kazuo Hara, Yasushi Yatabe, Yasuhiro Shimizu, Kei Muro, Takeshi Morimoto, Seiichi Hirota, Kiron M. Das, Hiroto Miwa

**Affiliations:** 1 Division of Gastroenterology, Department of Internal Medicine, Hyogo College of Medicine, Nishinomiya, Japan; 2 Department of Clinical Oncology, Aichi Cancer Center Hospital, Nagoya, Japan; 3 Department of Gastroenterological Surgery, Aichi Cancer Center Hospital, Nagoya, Japan; 4 Department of Endoscopy, Aichi Cancer Center Hospital, Nagoya, Japan; 5 Department of Gastroenterology, Aichi Cancer Center Hospital, Nagoya, Japan; 6 Department of Pathology and Molecular Diagnosis, Aichi Cancer Center Hospital, Nagoya, Japan; 7 Department of Clinical Epidemiology, Hyogo College of Medicine, Nishinomiya, Japan; 8 Department of Surgical Pathology, Hyogo College of Medicine, Nishinomiya, Japan; 9 Division of Gastroenterology and Hepatology, Departments of Medicine and Pathology, Robert Wood Johnson Medical School, Rutgers, Cancer Institute of New Jersey, New Brunswick, NJ USA

**Keywords:** Gastrointestinal cancer, Gastrointestinal cancer, Risk factors, Molecular medicine, Prognostic markers

## Abstract

Non-ampullary duodenal adenocarcinoma (NADC) is extremely rare. Little is known about its clinicopathological and molecular features or its management. Herein we retrospectively analyzed the cases of 32 NADC patients, focusing on microsatellite instability (MSI), genetic mutations, CpG island methylator phenotype (CIMP), and immunostaining including mucin phenotype and PD-L1 expression. The incidence of MSI, *KRAS*/*BRAF*/*GNAS* mutations and CIMP was 51.6%, 34.4%/3.1%/6.5% and 28.1%, respectively. PD-L1 expression was seen in 34.4% of patients. No significant associations between clinicopathological features and *KRAS*/*BRAF*/*GNAS* genetic mutations or CIMP were found. Histologically non-well-differentiated-type NADCs and those in the 1st portion of the duodenum were significantly associated with later stages (stages III–IV) (*P* = 0.006 and *P* = 0.003, respectively). Gastric-phenotype NADCs were frequently observed in the 1st portion and in late-stage patients; their cancer cells more frequently expressed PD-L1. Histologically, the non-well-differentiated type was an independent predictor of PD-L1 expression in cancer cells (OR 25.05, *P* = 0.04) and immune cells (OR 44.14, *P* = 0.02). Only late-stage disease (HR 12.23, *P* = 0.01) was a prognostic factor for worse overall survival in a Cox proportional hazards regression model. Our observation of high proportions of MSI and PD-L1 expression may prompt the consideration of immune checkpoint inhibitors as a new treatment option for NADCs.

## Introduction

Small bowel adenocarcinomas (SBAs) are rare, appearing at a rate of <1.0 per 100,000 people; when age-standardization is applied to the world population, the rate ranges from 0.3 to 2.0 per 100,000 people^1^. Duodenal adenocarcinoma (DAC), a type of SBA, represents <1% of all gastrointestinal (GI) cancers^2^, and the incidence of DAC is increasing worldwide^3,4^. Many studies regarding molecular events in SBAs and small intestinal adenomas have been reported^5–19^, but the associations among clinicopathological features, genetic/epigenetic alterations including CpG island methylation, and the phenotype (CIMP) and immunophenotype in DACs have not been clearly analyzed, unlike in the case of gastric and colorectal cancers (CRCs).

Because SBAs are so rare, little is known about their optimal management, including chemotherapy. No guidelines for the treatment of SBAs have been prepared by the U.S. National Comprehensive Cancer Network^20^. Clinical practice guidelines for the diagnosis, treatment and follow-up of SBA were recently published in France^21^. Although there have been four prospective studies on chemotherapy for SBA, no randomized trials of the efficacy of different chemotherapy regimens have been performed, and thus there is no established standard regimen for patients with unresectable or recurrent SBA^4^.

Le *et al*. reported that the microsatellite instability (MSI) status — defined as mismatch repair (MMR) deficiency — predicted the clinical benefit of immune checkpoint blockade in mainly CRCs, but they included only two cases of MMR-deficient SBAs in their analysis^22^. Several later studies investigated the association between the MSI status and the programmed cell death-ligand 1 (PD-L1) expression in GI cancers^22–27^. Immunotherapies using immune checkpoint inhibitors (ICIs) have demonstrated significant clinical benefits and a prolonged duration of response in subsets of patients with GI cancer^22–26^. Testing for biomarkers, including MSI and PD-L1, may therefore be necessary to broaden the identification of responders to ICI treatment and to achieve a better stratification of patients^27,28^.

We conducted the present study to retrospectively investigate the associations among the clinicopathological features, immunophenotype (including PD-L1 expression), and genetic or epigenetic alterations in non-ampullary duodenal adenocarcinomas (NADCs). We also evaluated whether those features — i.e., clinicopathological characteristics, immunophenotype, and/or molecular events — impact the survival of NADC patients.

## Results

### Clinicopathological characteristics in NADCs

The clinicopathological characteristics of the 32 patients with NADC are summarized in Table 1. The median age of the patients was 65.5 years (1st–3rd quartile 53–75 years), with women accounting for only 21.9% of the patients. The tumors were located mostly in the 2nd portion of the duodenum (65.6%), and the histologically well-differentiated-type adenocarcinoma was the most common type (75.0%). The tumor stages were 0–I (n = 18), II (n = 1), III (n = 9), and IV (n = 4).

**Table 1:** Clinicopathological and molecular characteristics of NADCs.

Characteristic	n	(%)
Mean age (1^st^–3^rd^ quartile) (yr)	65.5 (53–75)	
Gender		
Male	25	(78.1)
Female	7	(21.9)
Histology		
Well	24	(75.0)
Moderate	4	(12.5)
Poor	4	(12.5)
Tumor location		
1^st^ portion	8	(25.0)
2^nd^ portion	21	(65.6)
3^rd^ portion	3	(9.4)
Stage		
0–I	18	(56.3)
II	1	(3.1)
III	9	(28.1)
IV	4	(12.5)
**Immunohistochemistry**		
Mucin phenotype		
I–type	18	(56.3)
Mixed G-type	14	(43.8)
HER2 positive	0	(0)
Das-1 positive	24	(75.0)
PD-L1 positive (>1%)		
Tumor cell	6	(18.8)
Immune cell	11	(34.4)
MMR-deficiency^†^	8	(28.6)
**Molecular alterations**		
MSI^‡^	16	(51.6)
CIMP	9	(28.1)
*KRAS* mutated	11	(34.4)
*BRAF* mutated	1	(3.1)
*GNAS* mutated^‡^	2	(6.5)

^†^Four cases could not undergo immunohistochemistry due to an insufficient amount of material.

^‡^One sample in the MSI analysis and one sample in the *GNAS* mutation analysis could not be analyzed due to an insufficient amount of material.

CIMP: CpG island methylator phenotype; G-type: gastric type; HER2: human epidermal growth factor receptor type 2; I-type: intestinal type; MMR: mismatch repair; MSI: microsatellite instability; NADC: non-ampullary duodenal adenocarcinoma; PD-L1: programmed death ligand 1.

Mixed gastric (G)-type NADCs were identified in 14 cases (43.8%), comprising 3 G-type and 11 GI-type NADCs. The following expressions were observed: human epidermal growth factor receptor type 2 (HER2) (n = 0, 0%), Das-1 (n = 24, 75.0%), and PD-L1 (n = 11, 34.4%). When we evaluated the PD-L1 expression in cancer cells and immune cells in the stroma separately, the expression rate was 18.8% (6 of 32) in cancer cells and 34.3% (11 of 32) in immune cells. There was no case in which PD-L1 was expressed exclusively in cancer cells. MMR deficiency was seen in 8 of 26 patients (28.6%).

### Molecular alterations in the NADCs

Table 1 also shows the incidences of molecular events: 51.6% for MSI, 28.1% for CIMP and 34.4% for *KRAS* mutation. The incidences of *BRAF* and *GNAS* mutations were comparatively small. Insufficient amounts of DNA invalidated one MSI test and one *GNAS* mutation test. In the MSI analysis, a major pattern (as defined in the Methods section) was found in 8 of 31 patients (25.8%). Of the 11 (of 32; 34.4%) NADCs with *KRAS* mutations, GGT (Gly) changed to both GTT (Val) and GCT (Ala) (n = 1 case), both Val and CGT (Arg) (n = 3), both Ala and GAT (Asp) (n = 1), Asp (n = 2), AGT (Ser) (n = 1), Arg (n = 2), or Val (n = 1). *BRAF* mutation was detected in V600A in 1 patient: this NADC had MSI but did not have a *KRAS* mutation. *GNAS* mutations were detected in 2 cases: 1 case with c.602 G > A, and 1 case with c.602 G > G/A, both in codon 201 (R201H).

### Associations among the clinicopathological features and the immunohistochemical and molecular analysis results

The histologically non-well-differentiated-type (i.e., the moderately and poorly differentiated types) and tumors in the 1st portion of the duodenum were more frequently identified in the late stages (stages III–IV) (*P* = 0.006 and *P* = 0.003) in association with PD-L1 expression in cancer cells (*P* < 0.0001 and *P* = 0.02) and immune cells (*P* = 0.001 and *P* = 0.09), respectively. The late tumor stages were significantly associated with mixed G-type (*P* = 0.09) and PD-L1 expression in immune cells (*P* = 0.02). Additionally, mixed G-type tended to relate to PD-L1 expression in cancer cells (*P* = 0.06) (Table 2). However, other factors, i.e., monoclonal antibody (mAb) Das-1 reactivity, MSI, CIMP, and *KRAS* mutations, were not associated with clinicopathological features (Suppl. Table S3).

**Table 2:** Relationships among clinicopathological and molecular characteristics of NADCs.

	Histology				Tumor location			Tumor stage			Mucin phenotype		
	Well	Mod	Por	*P* ^†^	1^st^	2^nd^–3^rd^	*P*	0–II	III–IV	*P*	Mixed G-type	I-type	*P*
No. of patients	24	4	4		8	24		19	13		14	18	
Median age (yrs) (1^st^–3^rd^ quartile)	68.5 (61–75)	66 (47–85)	51 (47–72)	0.44^‡^	64.5 (54–77)	67.5 (53–75)	0.93	73 (61–75)	64 (49–72)	0.19	65.5 (59–73)	66.5 (51–77)	0.81
Male:Female	18:6	4:0	3:1	0.53	7:1	18:6	0.21	14:5	11:2	0.67	11:3	14:4	>0.99
Histology													
Well:Mod:Por	—	—	—		—	—	—	—	—	—	—	—	—
Tumor location													
1^st^:2^nd^–3^rd^	5:19	1:3	2:2	0.46	—	—	—	—	—	—	—	—	—
Tumor stage													
0–II:III–IV	18:6	0:4	1:3	0.006	1:7	18:6	0.003	—	—	—	—	—	—
Mucin phenotype													
Mixed G-type:I type	10:14	1:3	3:1	0.76	6:2	8:16	0.09	6:13	8:5	0.09	—	—	—
Das-1+:Das-1−	19:5	3:1	2:2	0.46	6:2	18:6	>0.99	15:4	9:4	0.68	10:4	14:4	0.70
PD-L1 expression													
Cancer cells +:−	2:22	0:4	4:0	<0.0001	4:4	2:22	0.02	2:17	4:9	0.19	5:9	1:17	0.06
Immune cells +:−	4:20	3:1	4:0	0.001	5:3	6:18	0.09	3:16	8:5	0.02	5:9	6:12	>0.99
MSI+:MSI−	10:14	3:1	3:0	0.098	5:3	11:12	0.69	9:10	7:5	0.55	6:7	10:8	0.61
CIMP+:CIMP−	6:18	1:3	2:2	0.58	2:6	7:17	>0.99	6:13	3:10	0.70	2:12	8:10	0.12
*KRAS*+:*KRAS*−	8:16	2:2	1:3	0.74	1:7	10:14	0.21	5:14	6:7	0.25	5:9	6:12	>0.99
*BRAF*+:*BRAF*−	1:23	0:4	0:4	0.84	0:8	:23	>0.99	1:19	0:13	>0.99	0:14	1:18	>0.99
*GNAS*+:*GNAS*−	1:22	0:4	1:3	0.26	1:7	1:22	0.46	0:19	2:10	0.14	2:12	0:18	0.18

^†^Well-differentiated type vs. moderately and poorly differentiated types.

^‡^Kruskal-Wallis test. Abbreviations are explained in the Table 1 footnote.

CIMP: CpG island methylator phenotype., G-type: gastric type, I-type: intestinal type, MSI: microsatellite instability, NADC: non-ampullary duodenal adenocarcinoma, PD-L1: programmed cell death-ligand 1.

PD-L1 expression was associated with histology (the non-well-differentiated type), tumor location (the 1st portion of the duodenum), late tumor stage, and mixed G-type. Although the number of PD-L1-positive cases was small, when we evaluated the expression in cancer cells and immune cells separately, only the non-well-differentiated type was a predictive factor of PD-L1 expression in both cancer cells (odds ratio [OR] 25.05, 95% confidence interval [CI]: 1.22–513.85, *P* = 0.04) and immune cells (OR 44.14, 95%CI: 1.96–995.97, *P* = 0.02) (Table 3).

**Table 3:** Relationship between clinicopathological/molecular characteristics and PD-L1 expression in cancer cells and immune cells.

	Cancer cell						Immune cell					
	PD-L1+	PD-L1−	*P*	Multivariate logistic analysis			PD-L1+	PD-L1−	*P*	Multivariate logistic analysis		
				OR	95% CI	*P*				OR	95% CI	*P*
No. of patients	6	26					11	21				
Median age (yrs) (1^st^–3^rd^ quartile)	56 (50–69)	70 (61–75)	0.17				61 (42–90)	71 (43–83)	0.20			
Male:Female	4:2	21:5					8:3	17:4				
Histology												
Well:Non-well	2:4	22:4	<0.0001	25.05	1.22–513.85	0.04	4:7	20 :1	0.001	44.14	1.96–995.97	0.02
Tumor location												
1^st^:2^nd^–3^rd^	4:2	4:22	0.02	9.60	0.65–141.71	0.09	5:6	3:18	0.09	7.03	0.40–123.82	0.18
Tumor stage												
0–II:III–IV	2:4	17:9	0.19				3:8	16:5	0.02	1.13	0.07–19.23	0.93
MSI+:MSI−	3:2	13:13	>0.99				6:4	10:11	0.70			
CIMP+:CIMP−	2:5	7:19	>0.99				4:7	5:16	0.68			
Mucin phenotype												
Mixed G-type:I-type	5:1	9:17	0.06	12.66	0.53–302.67	0.12	5:6	9:12	>0.99			
Das-1+:Das-1−	4:2	20:6	0.62				8:3	16:5	>0.99			
*KRAS*+:*KRAS*−	1:5	10:16	0.64				3:8	8:13	0.70			
*BRAF*+:*BRAF*−	0:6	1:25	>0.99				0:11	1:20	>0.99			
*GNAS*+:*GNAS*−	1:5	1:24	0.35				1:10	1:19	>0.99			

CIMP: CpG island methylator phenotype; G-type: gastric type; HER2: human epidermal growth factor receptor type 2; I-type: intestinal type; MMR: mismatch repair; MSI: microsatellite instability; NADC: non-ampullary duodenal adenocarcinoma; PD-L1: programmed death ligand 1.

Among the 19 NADCs with MSI, 7 cases (36.8%) stained positive for PD-L1 expression; there was no significant association between MSI and PD-L1 expression (*P* = 0.70, Suppl. Table S4).

### Survival analysis

Seventeen of the 19 patients in early stages (stages 0–II) were alive without disease at the last contact (range 6–110 months; mean 48 months). Of the remaining 2 cases, one patient died suddenly 3 months after the last visit, and the other patient died from the disease. The patients with late-stage disease (stages III–IV, n = 13) were alive at last contact (n = 2) or died of NADC (n = 11).

**Figure 1:**
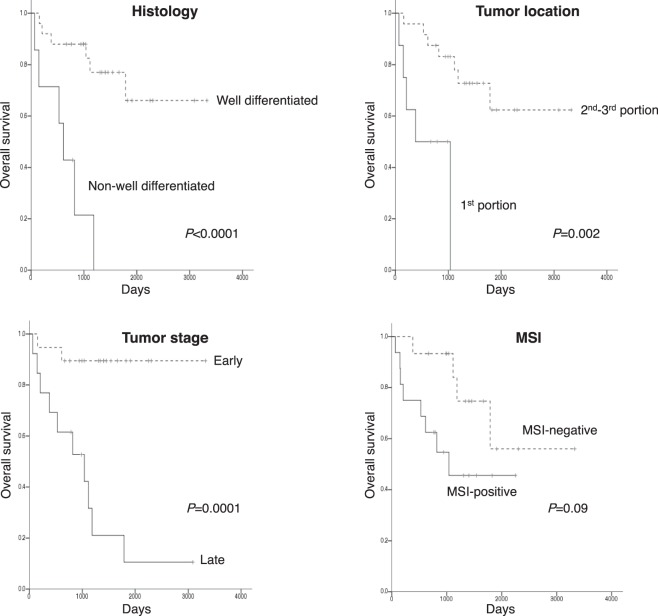
Kaplan-Meier survival curves of NADCs according to clinicopathological features and MSI. Non-well-differentiated-type histology (**A**), tumor location in the 1st portion (**B**), late tumor stage (stages III–IV) (**C**), and MSI positivity were associated with worse overall survival. *P*-values: log-rank test.

The Kaplan-Meier survival analysis demonstrated that the NADCs of the histologically non-well-differentiated type (*P* < 0.0001), in the 1st portion of the duodenum (*P* = 0.002), at the late stages (*P* = 0.0001), with MSI (*P* = 0.09), and with PD-L1 expression in immune cells (*P* = 0.05) were associated with worse overall survival (OS) by the log-rank test (Fig. 1), while there were no significant associations between other clinicopathological or molecular features and OS (Table 4, Suppl. Fig. S4). In the multivariate Cox proportional hazards regression model, the late tumor stages (HR 12.23, 95%CI: 1.67–134.56, *P* = 0.01) were independently associated with worse OS (Table 4).

**Table 4:** Relationship between clinicopathological/molecular characteristics and overall survival.

	Univariate analysis			Multivariate analysis		
	HR	95% CI	*P* (Cox)	HR	95% CI	*P*
Histology (non-well diff.- type *vs* well diff. -type)	8.16	2.36–29.49	0.001	1.61	0.07–4.57	0.64
Tumor location (1^st^ *vs* 2^nd^–3^rd^)	6.73	1.72–28.28	0.007	1.61	0.10–3.30	0.58
Mucin phenotype (mixed G-type *vs* I-type)	1.27	0.40–4.34	0.69			
Tumor stage (late *vs* early)	10.87	2.36–59.09	0.0002	12.23	1.67–134.56	0.01
PD-L1 expression in cancer cells (positive *vs* negative)	1.22	0.19–4.76	0.80			
PD-L1 expression in immune cells (positive *vs* negative)	2.99	0.91–9.79	0.07	1.52	0.23–9.41	0.65
MSI (positive *vs* negative)	2.73	0.86–10.41	0.09	4.10	0.69–33.12	0.12
CIMP (positive *vs* negative)	0.99	0.22–3.33	0.99			
*KRAS* (mutation *vs* wild type)	1.73	0.54–5.54	0.35			

CIMP: CpG inland methylator phenotype, G-type: gastric type, I-type: intestinal type, MSI: microsatellite instability, PD-L1: programmed cell death-ligand 1.

## Discussion

Prior studies on molecular events in NADCs have focused on genetic events^7,10,13–18,28^, and there have been few studies evaluating epigenetic alterations^6,9,12,16^. There have also been no studies of the associations among clinicopathological, immunohistochemical (including PD-L1 expression) and molecular characteristics; our study is the first to explore these associations in NADC, although a single study evaluated the associations in SBA^27^.

Herein we observed that the NADCs of the histologically moderately and poorly differentiated type (i.e., the non-well-differentiated type) and those in the 1st portion of the duodenum were significantly associated with late tumor stages (stages III–V). Mixed G-type was frequently identified in the late stages. Several studies have shown that duodenal tumors with a G-type component are associated with high histological atypia, location in the 1st portion of the duodenum^29–31^, and reduced disease-free survival^29^. Therefore, taking into consideration the past and present findings, we speculated that mixed G-type NADCs of histologically non-well-differentiated type in the 1st portion may be more likely to progress. Our analyses also revealed that late tumor stages were independently associated with worse OS, confirming that tumor stage is the most important prognostic factor in SBAs^4,7,11,32^.

PD-L1 expression in NADCs has not been described other than in two studies of ampulla of Vater carcinoma and SBA^19,27^; according to the findings of those studies, PD-L1 was expressed in 26.9–44% of duodenal cancers (an incidence that is similar to our present result). Many studies of PD-L1 evaluated its expression in both neoplastic cells and immune cells^19,27,33–35^, revealing that PD-L1 is more frequently expressed in immune cells than in neoplastic cells. Our present findings showed that there was no positivity of PD-L1 in cancer cells without positivity in immune cells, as in previous reports^27,33,34^.

The MSI rate in our study was higher (51.6%) than the reported rates in SBAs (7.6–33.3%)^5,7,8,11,13,14,18,19^. One of the explanations for this discrepancy may be differences in the methods of MSI analysis—i.e., differences in the immunohistochemistry for MMR proteins, the method of analysis (either polyacrylamide/urea gel electrophoresis following silver staining or next-generation sequencing), and the number and location of MSI markers evaluated. We identified MMR deficiency in 28.6% of the patients in our series, which is similar to the incidence of the major pattern (25.8%, 8 of 31) in our MSI analysis (as described in the Methods section below). The high-resolution fluorescent microsatellite analysis (HRFMA) assay used in the present study allows for a more accurate assessment of phenomena such as the allele shift pattern (minor pattern) compared to previous formats^36,37^. It might therefore be impossible to detect the minor pattern in MSI by using other methods^36,37^. In the future, it will be necessary to establish the definition of MSI in the HRFMA assay and other procedures. This lack of an established definition may be another cause of the discrepancy in the incidence between MSI and MMR deficiency in our study, although the incidence obtained by these two tests is considered to be similar in GI cancers^7,38^.

In general, the MSI tumor microenvironment strongly expresses several immune checkpoint ligands (including PD-L1), indicating that the active immune microenvironment of the tumors is counterbalanced by immune inhibitory signals that resist tumor elimination^39^. There are several reports regarding the association between MSI and PD-L1 expression in SBAs and other GI cancers^19,22,27,28,33,35,39,40^; some of these reports found a positive correlation between MSI and PD-L1 expression^19,22,28,39^ in most of GI cancers examined, except in SBA in one study^28^.

The lack of a positive association between MSI and PD-L1 expression in NADC in our present report (Suppl. Table S3) is thus in agreement with a recent report on some GI cancers including SBAs^28^. Generally, MSI cancers are associated with a higher mutational burden and tumor neoantigen load; these tumors provoke an antitumor immune response by dense immune cell infiltration, and thereby exhibit heightened sensitivity to ICIs^22,39^. Therefore, taking into account both these studies and our present findings, ICIs could be a new treatment option for NADCs that have MSI and/or are positive for PD-L1, as is the case in other cancers^22–24^.

In regard to the CIMP in SBAs, including DACs, there have been only a few studies^8,9,16^. Incidences of 26.6–29.7% were seen in those studies; our current result (28.1%) is within that range. However, CIMP was associated with MSI in those studies, contrary to our present finding. According to the studies analyzing duodenal adenomas and DACs^9,12^, CIMP is an early event in tumorigenesis and may be lost in later stages of tumor progression; alternatively, advanced-stage DACs may arise from different mechanisms.

It has also been reported that patients with CIMP-positive DACs showed worse OS than CIMP-negative cases^9^. We did not observe this association in the present study. The discrepancy may be due in part to differences in the analysis methods of CIMP and MSI mentioned above and in our previous investigations^33–35,37^.

The *KRAS* mutation rate we observed among 32 NADCs (34.4%) was in line with those in previous reports^9–11,13,15,16^ except for one study (11%)^14^. Fu *et al*. reported that the *KRAS* G > A mutation correlated significantly with late-stage disease and poor tumor differentiation^10^. In our present investigation however, there were only two cases with *KRAS* G > A mutation: the well-differentiated type and stage 0 in one case and the moderately differentiated type and stage III in the other. In addition, *KRAS* mutation was not related to the mucin phenotype as reported by Matsubara *et al*.^15^.

There have been only a few reports of *BRAF* and *GNAS* mutations in NADCs, and *BRAF* and *GNAS* mutations were as rare in our patients as in those studies^13–15,17^. Warth *et al*. showed that *BRAF* mutations were associated with SBAs with CIMP, but they did not separately analyze NADC data^8^. Another interesting study reported that *GNAS* mutations are more common in DACs with the G-type phenotype^15^. We did not observe this association in our present analyses; further studies with larger sample sizes are needed to clarify the reason for this lack of association.

We previously reported that all SBAs (8 of 8) reacted highly with the mAb Das-1^41^, which is in agreement with our present findings for NADCs. Das-1 specifically reacts with the colonic epithelium and not with enterocytes (including goblet cells) from the jejunum or ileum^42^. The high reactivity of Das-1, a unique epitope related to colonic metaplasia, may therefore indicate that there is a phenotypic change of small intestinal enterocytes to colonocytes in NADCs. We detected no HER2 expression, as was the case in previous studies^4,11^. However, two studies that evaluated a large number of SBCs identified *HER2* genomic alteration in 8.4–9.5% of their patients^13,18^.

It has generally been considered that in gastric cancer and CRC, tumors with MSI have better survival than those without^43,44^, but our present findings contradict that assumption. The association between MSI and overall survival in DAC remains uncertain. Overman *et al*. provided evidence of an improved prognosis in a subset of patients with a deficient expression of MMR proteins, which indicates MSI^7^, whereas Aparicio *et al*. reported that DAC with MMR deficiency was associated with longer OS^11^. Future investigations with larger sample sizes are needed to more clearly elucidate the relationship between MSI and survival in NADC patients.

The present study has some limitations. First, this was a retrospective analysis of a relatively small number of NADC patients, particularly considering that several different cellular phenotypes and molecular markers were evaluated. Second, we did not evaluate the CD8+ T-cell density in intratumoral or immune stroma. It was recently reported that increasing CD8+ densities in both tumors and immune stroma were associated with increasing percentages of tumor and stromal PD-L1 expression, indicating adaptive immune resistance^45^. An additional study is needed to evaluate the integration of biomarkers such as MSI and PD-L1 with CD8+ tumor-infiltrating lymphocytes.

In conclusion, although the number of NADC cases investigated was small (n = 32), our results suggest that in NADCs, genetic (*KRAS*/*BRAF*/*GNAS* mutations) and epigenetic alterations (CIMP) are not involved in the clinicopathological characteristics. However, MSI was more frequently observed and was significantly associated with clinical behavior, and the histology and tumor location were involved in the late tumor stage. Notably, histologically non-well-differentiated-type NADCs were an independent predictor of PD-L1 expression in both cancer and immune cells, but MSI was not. Since NADCs have molecular alterations that are different from those in gastric cancer and CRC, other therapeutic strategies may be necessary. Our results may thus indicate that ICIs could be a promising novel treatment for NADCs.

## Patients, Materials, and Methods

### Patients

Thirty-two consecutive patients with NADC treated at Hyogo College of Medicine Hospital or Aichi Cancer Center Hospital between April 2009 and March 2016 were enrolled in this study. There were no patients with a family history of cancer. Formalin-fixed, paraffin-embedded (FFPE) tissue blocks from patients who had been biopsied or resected by endoscopy or surgery for stage 0–IV NADC were obtained. The tissue sections from all cases in this study were reviewed by expert GI pathologists (S.H. and Y.Y.).

### Consent and institutional review board approval

The study was approved by the Institutional Review Boards of Hyogo College of Medicine (No. Rin-Hi 315) and Aichi Cancer Center Hospital (No. 2016-1-090). This trial was registered with the UMIN Clinical Trials Registry (No. UMIN000023622). The informed consent of each patient was obtained by the opt-out procedure or as written informed consent, according to the procedure described in the study protocol (Rin-Hi 315 and 2016-1-090). The study was performed in accordance with the Declaration of Helsinki.

### Clinicopathological and tumor evaluations

We evaluated the patients’ clinical data for both demographics (age and sex) and tumor characteristics (histology and tumor location). Their NADCs were staged using the combined American Joint Committee on Cancer/International Union Against Cancer (AJCC/UICC) TNM staging system^46^.

### Immunohistochemistry

FFPE tissue blocks were cut into 4-µm-thick tissue sections and subjected to both hematoxylin and eosin staining and immunohistochemical staining. For immunostaining, the avidin-biotin peroxidase complex method was used to detect MUC5AC (CLH2; Agilent Technologies, Santa Clara, CA), MUC6 (CLH5, 1:50; Leica Biosystems, Newcastle, UK), MUC2 (clone Ccp58; Agilent Technologies), and CD10 (clone 56C6; Agilent Technologies). Immunohistochemical staining for gastric phenotype markers (MUC5AC and MUC6) and intestinal phenotype markers (MUC2 and CD10) was considered positive when distinct staining was observed in >10% of the cancer cells, as described previously^29^.

On the basis of the mucin histochemistry, the NADCs were classified into three subtypes: (1) gastric phenotype (G-type), (2) gastric and intestinal phenotype (GI-type), and (3) intestinal phenotype (I-type)^29^. NADCs that showed immunoreactivity for either the G-type or the G-I type were defined as mixed G-type.

HER2 testing was performed for selected cases using the HER2 monoclonal antibody (mAb) (4B5; Ventana Medical Systems, Tucson, AZ) with an automated slide stainer (BenchMark XT; Ventana Medical Systems) per the manufacturer’s protocol. The staining for HER2 was graded according to the guideline for HER2 testing in cancer cells, with 2+ or 3+ membranous staining in >10% of the tumor cells counted as positive^47^.

**Figure 2:**
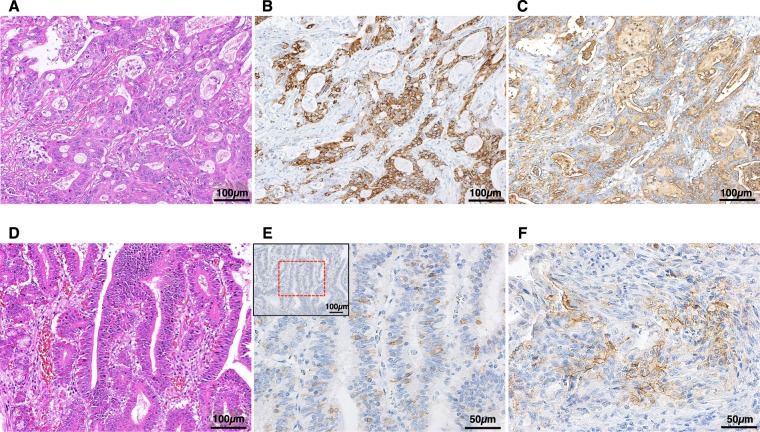
Staining of the serial sections of NADCs. (**A**) Hematoxylin and eosin (H&E) staining shows moderately differentiated-type adenocarcinoma (×200). NADC strongly reacted with MUC5AC (**B**, ×200) and the mAb Das-1 (**C**, ×200). (**D**) H&E staining revealed well differentiated-type adenocarcinoma. (**E**) *Inset* at the upper left: PD-L1 staining of (D) (×200). PD-L1 is expressed in cancer cells, as outlined in part by a *red square* (×400). (**F**) Immune cells were also positive for PD-L1 expression (×400).

The mAb Das-1, which is highly specific against the colonic phenotype, is considered clinically useful in identifying small intestinal adenomas at “high risk” for malignancy^41^. Therefore, mAb Das-1 staining was also performed using sensitive immunoperoxidase assays, as described in previous reports^41,48–50^. Positive expression was defined as >10% of tumor cells staining for mAb Das-1^49,50^ (Fig. 2C).

For the PD-L1 staining, sections were retrieved in EDTA buffer (pH 8.0) at 98 °C for 20 min. The immunostaining used a mAb against the cytoplasmic domain of PD-L1 (clone E1L3N, dilution 1:400; Cell Signaling Technology, Danvers, MA), and the reactivity was evaluated separately for cancer cells and infiltrating immune cells. PD-L1 positivity was defined as positive cell staining of any intensity on ≥1% of the cell membrane. Cytoplasmic staining was not considered in this study^24,33^ (Fig. 2E,F).

The expression of MMR proteins such as MLH1 (ES05, 1:50), MSH2 (79H11, 1:50), MSH6 (PU29, 1:40) and PMS2 (M0R4G, 1:10; Leica Biosystems) was also examined. Regarding the criteria for MMR protein expression, if nuclear staining was identified in the cancerous cells, the lesion was defined as positive for that marker. Positive staining for all of these proteins was regarded as proficient MMR, and negative staining for any of these four proteins was regarded as deficient MMR^51^. In this study, four cases—i.e., two biopsy samples and two endoscopically resected specimens—could not be evaluated by the immunohistochemistry of MMR proteins because of an insufficient amount of material.

### DNA extraction

Two or three 7-µm-thick tissue sections were cut for DNA extraction for a molecular analysis. DNA was extracted from the NADC and non-neoplastic normal mucosa using a QIAamp DNA Micro Kit (Qiagen, Hilden, Germany). The NADC and normal mucosa were isolated using a PALM MicroBeam LCM system (Microlaser Technologies, Munich, Germany) as described^48–50^.

### Analysis of MSI by HRFMA

For the high-resolution fluorescent microsatellite analysis (HRFMA) following reported methods^36,37,48–51^, we examined the following five microsatellite loci on chromosomes for MSI based on the revised Bethesda panel^52^: BAT26, BAT25, D2S123, D5S346, and D17S250. The MSI status was judged as described previously^36,37,49–51^. There were two MSI patterns: (1) unequivocal extra peak bands in tumor DNA that differed by a multiple of two base pairs (bp) in dinucleotide markers or one bp in mononucleotide markers from DNA in normal DNA (minor pattern) (Suppl. Fig. S1A), and (2) the appearance of a large number of additional alleles in the tumor DNA (major pattern) (Suppl. Fig. S1B,C), as described previously^36,37^.

In cases in which MSI and loss of heterozygosity were indistinguishable^53^, we calculated the allelic imbalance (AI) ratio. We considered MSI as positive when the AI ratio (normal allele 1: normal allele 2 or tumor allele 1: tumor allele 2) was <0.67 or >1.35, as reported previously^49–51^ (Suppl. Fig. S1D). The lesions were defined as having MSI when unstable loci were observed in two or more of the five investigated markers^37,49–51^. One sample in the MSI test could not be analyzed due to an insufficient amount of DNA.

### KRAS, BRAF, and GNAS mutation analysis

*KRAS* mutations in codons 12 and 13 were analyzed using a Mutector™ mutation detection kit (TrimGen, Sparks, MD) (Suppl. Fig. S2A), and the analysis of *BRAF* mutations in V600 was performed using a Mutector™ II (TrimGen) kit as reported previously^51^ (Suppl. Fig. S2B). These processes, involving a series of mutation analyses, were performed in accordance with the manufacturer’s instructions. *GNAS* mutation in exon 8 was also analyzed.

The polymerase chain reaction (PCR) products were electrophoresed on a 2% (w/v) agarose gel and recovered using a QIAquick Gel Extraction Kit (Qiagen). The isolated PCR products were sequenced using a Genetic Analyzer (3130xl; Applied Biosystems, Foster, CA) (Suppl. Fig. S2C). One *GNAS* mutation case could not be analyzed due to an insufficient amount of DNA.

### Sodium bisulfite modification of DNA and CIMP markers

As in earlier studies^48–51^, purified DNA samples were chemically modified by sodium bisulfite with an EpiTect^®^ Fast Bisulfite Kit (Qiagen). The bisulfite-modified DNA was amplified using primer pairs that specifically amplify the methylated or unmethylated sequences of several genes/loci related to carcinogenesis, including *CDH1*, *CDKN2A*, *MLH1*, MINT1, MINT31, *MGMT*, and *RUNX3*. These genes were used as CIMP markers.

### Methylation-sensitive high-resolution melting analyses

We performed a methylation-sensitive high-resolution melting (MS-HRM) analysis as described previously^48–51^. Briefly, PCR amplification and the MS-HRM analysis were performed using a LightCycler^®^ 480 System II (Roche, Mannheim, Germany). The primer sequences of all genes for the methylated and unmethylated forms and the PCR and MS-HRM conditions are summarized in Supplementary Tables S1 and S2. The percentages of methylation (0%, 10%, 50%, and 100%) were used to draw the standard curve (Suppl. Fig. S3). In this study, only samples with >10% methylation were considered methylated^48–51^. Samples in which ≥3 of the 7 CIMP panel markers were methylated were considered positive for CIMP^49–51^.

### Statistical analyses

Categorical variables are presented as numbers and percentages and were compared by the chi-square test between groups or Fisher’s exact test when appropriate. Continuous variables are expressed as the median and interquartile range and were compared by the Kruskal-Wallis test or Mann-Whitney U*-*test between groups. The results of univariate analyses of the clinicopathological features, immunophenotype and genetic or epigenetic alterations (including CIMP) were evaluated using logistic regression models to calculate crude ORs and 95%CIs. We used multivariate logistic regression models with forward variable selections to calculate adjusted ORs for significant factors when a *P*-value < 0.1 was identified in the univariate analysis. Differences at *P* < 0.05 were considered significant.

We constructed OS curves using the Kaplan-Meier method, and we used the log-rank test to evaluate the statistical significance of differences and associations with each clinicopathological or molecular marker. The Cox proportional hazards regression model was used to assess the predictive effects of multiple covariates (including histology, tumor stage, immunohistochemical results, and molecular events) on the OS simultaneously. All statistical analyses were performed using JMP Pro 13 (SAS Institute, Cary, NC) and SPSS 22.0 (SPSS, Chicago, IL).

## Supplementary information

Supplementary Figures and Tables
