# Supplementary material for: Molecular alterations and PD-L1 expression in non-ampullary duodenal adenocarcinoma: Associations among clinicopathological, immunophenotypic and molecular features
Source: Sci Rep. 2019 Jul 19;9:10526. doi: 10.1038/s41598-019-46167-y (PMC6642201; doi:10.1038/s41598-019-46167-y)
Supplement: Supplementary file 1 — Supplementary Figures and Tables [file 41598_2019_46167_MOESM1_ESM.docx]

**Molecular alterations and PD-L1 expression in non-ampullary duodenal adenocarcinoma: Associations among clinicopathological, immunophenotypic and molecular features**

Jiro Watari ^1^, Seiichiro Mitani ^2^, Chiyomi Ito ^1^, Katsuyuki Tozawa ^1^, Toshihiko Tomita ^1^, Tadayuki Oshima ^1^, Hirokazu Fukui ^1^, Shigenori Kadowaki ^2^, Seiji Natsume ^3^, Yoshiki Senda ^3^, Masahiro Tajika ^4^, Kazuo Hara ^5^, Yasushi Yatabe ^6^, Yasuhiro Shimizu ^3^, Kei Muro ^2^, Takeshi Morimoto ^7^, Seiichi Hirota ^8^, Kiron M. Das ^9^ and Hiroto Miwa ^1^

^1^ Division of Gastroenterology, Department of Internal Medicine, Hyogo College of Medicine, Japan; ^2^ Department of Clinical Oncology, Aichi Cancer Center Hospital, Japan; ^3^ Department of Gastroenterological Surgery, Aichi Cancer Center Hospital, Japan; ^4^ Department of Endoscopy, Aichi Cancer Center Hospital, Japan; ^5^ Department of Gastroenterology, Aichi Cancer Center Hospital, Japan; ^6^ Department of Pathology and Molecular Diagnosis, Aichi Cancer Center Hospital, Japan; ^7^ Department of Clinical Epidemiology, Hyogo College of Medicine, Japan; ^8^ Department of Surgical Pathology, Hyogo College of Medicine, Japan; ^9^ Division of Gastroenterology and Hepatology, Departments of Medicine and Pathology, Robert Wood Johnson Medical School, Rutgers, Cancer Institute of New Jersey, New Brunswick, United States

**Supplementary Figure S1.** Examples of MSI in NADC by high-resolution fluorescent microsatellite analysis. (A) The MSI on BAT25 is seen as an unequivocal extra peak shift compared with the control. (B) MSI on BAT25 is identified by the appearance of multiple additional peaks (arrows). (C) The MSI on D5S346 is identified by the appearance of multiple additional peaks (arrows) as a new allele. (D) T1 and N1 represent the highest respective peak areas of the shorter allele in NADC, and control samples and T2 and N2 represent the highest respective peak areas of the longer allele. MSI on D2S123 (longer allele, T2) of NADC is seen. The allelic imbalance ratio was 3.26.

**Supplementary Figure S2.** Representative mutations of the *KRAS* and *BRAF* genes determined by a shifted termination assay and *GNAS* mutation in exon 8 by sequencing. (A) A *KRAS* mutation was detected in codon 12, and the mutation pattern consisted of a transition to AGT (Ser) from GGT (Gly). (B) The mutation pattern of the *BRAF* gene was revealed to be the V600A pattern. (C) An identical missense mutation in the *GNAS* gene is present (arrowhead). *GNAS* was sequenced using a reverse primer.

**Supplementary Figure S3.** Representative results of MS-HRM analysis for methylation. Results are shown for the MINT31 locus, with positivity (full methylation) and negative controls (complete lack of methylation). The melting peaks were calculated from melting curves of MS-HRM. Each sample was directly compared with its control to identify the sample’s methylation status, and the differences in fluorescence between samples were normalized by the analysis algorithms. Methylated and partially methylated DNA (≥ 10%) were both considered to be positive for methylation; only completely unmethylated DNA was treated as negative. The sample shows a moderate level of methylation (≥ 50%).

**Supplementary Figure S4.** Kaplan-Meier survival curve according to immunohistochemical features, i.e., mucin phenotype and PD-L1 expression, and *KRAS* gene mutation in NADCs. No associations were observed between either the mucin phenotype, PD-L1 expression in cancer cells or immune cells, or *KRAS* mutation and the overall survival.

Supplementary Table S1. Primer sequences for the *GNAS* mutation analysis and MS-HRM assays

| Gene | Primer sequences (5’ – 3’) |
| --- | --- |
| *GNAS* | F - GACTATGTGCCGAGCGATCA |
| (Exon 8) | R - CACGTCAAACATGCTGGTGG |
| *CDH1* | F - GGAATTGTAAAGTATTTGTGAGTTTG |
|  | R - AAAATACCTTCAACCAATCACCTC |
| *CDKN2A* | F- CGGAGGAAGAAAGAGGAGGGGT |
|  | R- CGCTACCTACTCTCCCCCTCT |
| *MLH1* | F - TTGGTATTTAAGTTGTTTAATTAATAGTTG |
|  | R - AAAATACCTTCAACCAATCACCTC |
| *MGMT* | F- GTTTCGGGTTTCGTATTTATTTTGAAGG |
|  | R- GACAACCCCAACTTCCTCTACTC |
| MINT1 | F- GGGGTTGAGGTTTTTTGTTAG |
|  | R- AATCCCTCTCCCCTCTAAACTT |
| MINT31 | F- GGGTGATGGTTTTAGTAAAGTGAG |
|  | R- AAAAACACTTCCCCAACATCTAC |
| *RUNX3* | F- GTTTCGGGTTTCGTATTTATTTTGAAGG |
|  | R- GACAACCCCAACTTCCTCTACTC |

**Supplementary Table S2.** PCR and MS-HRM conditions in each gene

| Pre-incubation | | |  |  | Amplification | | | | | |  |  | Cooling | | |
| --- | --- | --- | --- | --- | --- | --- | --- | --- | --- | --- | --- | --- | --- | --- | --- |
| Denaturation (°C) | Hold  (min) | Ramp rate  (°C/s) |  |  | Target  (°C) | Hold  (sec) | Ramp rate  (°C/s) | Sec target^*^  (°C) | Step size^*^  (°C) | Step delay^*^  (°C) | Cycles |  | Target  (°C) | Hold  (sec) | Ramp rate  (°C/s) |
| 95 | 10 ^a,b,c,d,e,f^  15 ^g^ | 4.8 |  | Denaturation | 95 | 10 ^c,d,e,f^  15 ^g^  30 ^a,b^ | 4.8 |  |  |  | 45 ^a,b,d,e^  50 ^c,f,g^ |  | 40 | 30 | 2.5 |
|  |  |  |  | Annealing | 51 ^d^, 55 ^g^  62 ^e^, 63 ^b^  64 ^c^, 65 ^a,f^ | 10 ^d,e^  15 ^g^  30 ^a,b,c,f^ | 2.5 | 50 ^e^, 52 ^c^  53 ^f^, 57 ^b^  60 ^a^ | 0.5 ^a,b,c,e,f^ | 1 ^a,b,c,e,f^ |  |  |  |  |  |
|  |  |  |  | Extension | 72 | 10 ^d,e^  15 ^b^  20 ^g^  30 ^a,c,f^ | 4.8 |  |  |  |  |  |  |  |  |
|  |  |  |  |  |  |  |  |  |  |  |  |  |  |  |  |
|  |  |  |  |  | High-resolution melting | | | | | |  |  |  |  |  |
|  |  |  |  | Denaturation | 95 ^a,b,c,d,e,f^  97 ^g^ | 10 ^b^  60 ^a,c,d,e,f,g^ | 4.8 |  |  |  |  |  |  |  |  |
|  |  |  |  | Annealing | 40 ^a,c,d,e,f,g^  50 ^b^ | 60 | 2.5 |  |  |  |  |  |  |  |  |
|  |  |  |  | Melting interval | 65 | 1 ^a,c,d,e,f,g^  15 ^b^ | 4.8 |  |  |  |  |  |  |  |  |
|  |  |  |  | Continuous | 95 | - | 0.02 | Acquisition 25 (/°C) | | |  |  |  |  |  |

^*^ Touchdown method, ^a^ *CDH1*, ^b^ *CDKN2A*, ^c^ *MLH1,* ^d^ *MGMT*, ^e^ MINT1*,* ^f^ MINT31*,* ^g^ *RUNX3*

|  | Das-1 reactivity | | |  | MSI | | |  | CIMP | | |  | *KRAS* | | |
| --- | --- | --- | --- | --- | --- | --- | --- | --- | --- | --- | --- | --- | --- | --- | --- |
|  | + | – | *P* |  | + | – | *P* |  | + | – | *P* |  | + | – | *P* |
| No. of patients | 24 | 8 |  |  | 16 | 15 ^†^ |  |  | 9 | 23 |  |  | 11 | 21 |  |
| Median age (yr)  (1^st^–3^rd^ quartile) | 69  (57-75) | 63  (51-71) | 0.35 |  | 64  (51-78) | 69  (62-73) | 0.86 |  | 75  (58-81) | 64  (52-73) | 0.07 |  | 66  (51-75) | 65  (59-75) | 0.66 |
| Male : Female | 19 : 5 | 6 : 2 | > 0.99 |  | 13 : 3 | 11 : 4 | 0.69 |  | 6 : 3 | 19 : 4 | 0.37 |  | 10 : 1 | 15 : 6 | 0.37 |
| Histology |  |  |  |  |  |  |  |  |  |  |  |  |  |  |  |
| Well : Mod : Por | 19 : 3 : 2 | 5 : 1 : 2 | 0.46 |  | 10 : 3 : 3 | 14 : 1 : 0 | 0.10 |  | 3 : 1 : 2 | 18 : 3 : 2 | 0.53 |  | 8 : 2 : 1 | 16 : 2 : 3 | 0.74 |
| Tumor location |  |  |  |  |  |  |  |  |  |  |  |  |  |  |  |
| 1^st^ : 2^nd^–3^rd^ | 6 : 18 | 2 : 6 | > 0.99 |  | 5 : 11 | 3 : 12 | 0.69 |  | 2 : 7 | 6 : 17 | > 0.99 |  | 1 : 10 | 7 : 14 | 0.21 |
| Tumor stage |  |  |  |  |  |  |  |  |  |  |  |  |  |  |  |
| 0–II : III–IV | 15 : 9 | 4 : 4 | 0.68 |  | 9 : 7 | 10 : 5 | 0.55 |  | 6 : 3 | 13 : 10 | 0.70 |  | 5 : 6 | 14 : 7 | 0.28 |
| Mucin phenotype |  |  |  |  |  |  |  |  |  |  |  |  |  |  |  |
| Mixed G-type : I type | 10 : 14 | 4 : 4 | 0.70 |  | 6 : 10 | 7 : 8 | 0.61 |  | 2 : 7 | 12 : 11 | 0.23 |  | 5 : 6 | 9 : 12 | > 0.99 |
| Das-1+ : Das-1– | – | – | – |  | 12 : 4 | 11 : 4 | > 0.99 |  | 7 : 2 | 17 : 6 | > 0.99 |  | 9 : 2 | 15 : 6 | 0.68 |
| PD-L1 expression | 8 : 16 | 3 : 5 |  |  | 6 : 10 | 4 : 11 |  |  | 4 : 5 | 7 : 16 |  |  | 3 : 8 | 8 : 13 |  |
| Cancer cells + : – | 4 : 20 | 2 : 6 | 0.62 |  | 3 : 13 | 2 : 13 | > 0.99 |  | 2 : 7 | 4 : 19 | > 0.99 |  | 1 : 10 | 5 : 16 | 0.64 |
| Immune cells + : – | 8 : 16 | 3 : 5 | > 0.99 |  | 6 : 10 | 4 : 11 | 0.70 |  | 4 : 5 | 7 : 16 | 0.68 |  | 3 : 8 | 8 : 13 | 0.70 |
| MSI+ : MSI– | 15 : 8 * | 4 : 4 | 0.68 |  | – | – | – |  | 6 : 2 | 13 : 10 | 0.43 |  | 6 : 4 * | 13 : 8 | > 0.99 |
| CIMP+ : CIMP– | 7 : 17 | 2 : 6 | > 0.99 |  | 6 : 10 | 2 : 13 | 0.22 |  | – | – | – |  | 3 : 8 | 6 : 15 | > 0.99 |
| *KRAS*+ : *KRAS*– | 9 : 15 | 2 : 6 | 0.68 |  | 5 : 11 | 5 : 10 | > 0.99 |  | 3 : 6 | 8 : 15 | > 0.99 |  | – | – | – |
| *BRAF*+ : *BRAF*– | 1 : 23 | 0 : 8 | > 0.99 |  | 1 : 15 | 0 : 15 | > 0.99 |  | 0 : 9 | 1 : 22 | > 0.99 |  | 0 : 11 | 1 : 20 | > 0.99 |
| *GNAS*+ : *GNAS*– | 2 : 22 | 0 : 7 * | > 0.99 |  | 1 : 15 | 0 : 14 * | > 0.99 |  | 1 : 8 | 1 : 21 | 0.50 |  | 1 : 9 * | 1 : 20 | > 0.99 |

Supplementary Table S3. The relationship between clinicopathological and molecular characteristics of NADCs

^†^ lack of DNA volume.

**Supplementary Table S4.** Association between MSI and PD-L1 expression

|  |  | MSI | |
| --- | --- | --- | --- |
|  |  | + | – |
| PD-L1 expression | + | 7 | 3 |
|  | – | 12 | 9 |
